# Recurrent Rhabdomyolysis Induced by Severe Hypothyroidism

**DOI:** 10.7759/cureus.4818

**Published:** 2019-06-03

**Authors:** Varvara Boryushkina, Samihah Ahmed, Kalimullah Quadri, Adesh Ramdass

**Affiliations:** 1 Internal Medicine, Northwell Health Mather Hospital, Port Jefferson, USA

**Keywords:** recurrent rhabdomyolysis, hypothyroidism, rhabdomyolysis complications, severe hypothyroidism

## Abstract

Hypothyroidism is frequently associated with myalgias, muscle stiffness, easy fatigability, and occasionally some degree of myopathy with mildly elevated muscle enzymes. Rarely, hypothyroidism may be complicated by rhabdomyolysis, the rapid destruction of skeletal muscle with myoglobin, creatine kinase, urate, and electrolytes release into the circulation. Recurrent cases of rhabdomyolysis are uncommon as most patients experience only one episode of rhabdomyolysis in their lifetime. Most common causes of such episodes are trauma, epileptic seizures, or medication.

We describe a case of a 49-year-old male with a history of hypothyroidism, who repeatedly developed severe rhabdomyolysis precipitated by deep muscle injury, seizure, and poor medication compliance. Interestingly, he never developed any of the complications of rhabdomyolysis despite high levels of serum creatine kinase.

The most common and feared complication of rhabdomyolysis is acute kidney injury which can occur in 15 to 50% of patients with rhabdomyolysis. Timely and appropriate fluid resuscitation is the mainstay therapy for acute kidney injury (AKI) prevention. Recurrent rhabdomyolysis in a patient should prompt further investigation if there is a family history of a neuromuscular disorder or exercise intolerance. In a case of refractory hypothyroidism, a patient should be counseled on proper regimen and medication compliance.

## Introduction

Hypothyroidism is frequently associated with myalgias, muscle stiffness, easy fatigability, and occasionally some degree of myopathy with mildly elevated muscle enzymes. Rarely, hypothyroidism may be complicated by rhabdomyolysis, the rapid destruction of skeletal muscle with the release of myoglobin, creatine kinase, urate, and electrolytes into the circulation. It is potentially life-threatening complication associated with acute renal failure, metabolic derangements, disseminated intravascular coagulation, compartment syndrome, and peripheral neuropathy [1].

Recurrent cases of rhabdomyolysis are rare as most patients experience only one episode of rhabdomyolysis in their lifetime. Most common causes of such episodes are trauma, epileptic seizures, or medication. Further investigation should be considered if there is a family history of exercise intolerance or neuromuscular disorders [2].

## Case presentation

A 49-year-old male with a medical history of hypothyroidism, polymyositis, seizure disorder, secondary adrenal insufficiency, and depression was admitted for scheduled surgical debridement of a traumatic thigh wound contracted after a fall. Three months prior, the patient fell after a seizure and had an impaled piece of wood in his left thigh. He had surgical removal and debridement at the time with a second surgical debridement scheduled at this admission to promote further healing.

Patient’s history was notable for hypothyroidism refractory to a high dose of oral levothyroxine therapy. He was diagnosed with hypothyroidism 10 years before presentation, was started on oral Synthroid 200 mcg but did not increase his serum thyroxine levels. The patient was tested with levothyroxine absorption challenge tests and deemed to have poor absorption. He required initiation of parenteral levothyroxine 500 mcg intramuscularly twice weekly. However, he admitted to poor compliance with levothyroxine injections overall and specifically for the preceding few weeks. In addition, the patient was diagnosed with Addison's disease several years before presentation. Subsequently, adrenocorticotropic hormone (ACTH) stimulation test was normal. The patient did not require daily steroids.

Patient's social history was negative for alcohol or substance use confirmed by negative urine toxicology on every admission.

The patient has had frequent hospitalization for the past two years, usually for seizures, post-ictal state or status post fall. On every presentation, his free thyroxine was less than 0.25 ng/dL, or undetectable, thyroid stimulating hormone (TSH) levels were fluctuating between 53.505 and 168.209 ulU/m, creatine kinase (CK) level was usually in a range of 2326-16445 U/L (Table 1). Despite these high numbers, the patient has never exhibited any signs of myxedema such as confusion, lethargy with obtundation, hypoventilation, or hypothermia.

**Table 1:** Laboratory data TSH: Thyroid stimulating hormone; CK: Creatine kinase; Cr: Creatinine.

Variable	Reference range	Occasion 1	Occasion 2	Occasion 3	Occasion 4	Occasion 5	Occasion 6	Occasion 7	Occasion 8
CK, U/L	55-170	11451	15697	13728	12621	5256	16445	6786	10808
TSH, ulU/m	0.34-4.0		160.565	180.448	155.810	148.594	120.604		189.152
Cr, U/L	0.7-1.2	1.3	1.5	1.4	1.3	1.4	1.3	1.5	1.5

At the time of admission for surgical debridement of the thigh wound, he complained of fatigue, weakness, generalized muscle pain and frequent recurrent falls. His last seizure was two weeks before presentation. Review of systems was significant for dry skin, weight gain over the past few months, constipation, and long-standing depression.

The patient had gained 14 lbs since he was seen last eight weeks ago. He was noted to have voice hoarseness, slow speech, pale coarse skin, edematous puffy face, periorbital edema, enlargement of the tongue, non-pitting edema of upper and lower extremities, delayed relaxation of deep tendon reflexes, muscle tenderness throughout, left upper thigh with wound, surrounding erythema and induration, with mild drainage and purulent exudate. The remainder of his physical examination was unremarkable.

Laboratory testing showed elevated serum CK of 10808 U/l, TSH of 189.152 uIU/m, triiodothyronine (T3) less than 0.5 ng/ml, and undetectable free thyroxine consistent with uncontrolled hypothyroidism complicated by severe rhabdomyolysis.

Other laboratory tests were consistent with hypothyroidism: normochromic normocytic anemia with hemoglobin of 10.3 g/dL and MCV of 89, hypertriglyceridemia at 617 mg/dL, mildly elevated liver enzymes - aspartate transaminase (AST) at 122 U/L and alanine transaminase (ALT) at 64 U/L. Markers for hepatitis C and B were negative. No electrolyte (sodium, potassium, phosphate, calcium) abnormalities were noted. In addition, the patient had mildly elevated creatinine at 1.5 mg/dL (baseline 1.2 mg/dL). Urinalysis was notable for the presence of hyaline casts, mild proteinuria without microscopic hematuria. Urine myoglobin were not measured.

**Figure 1:**
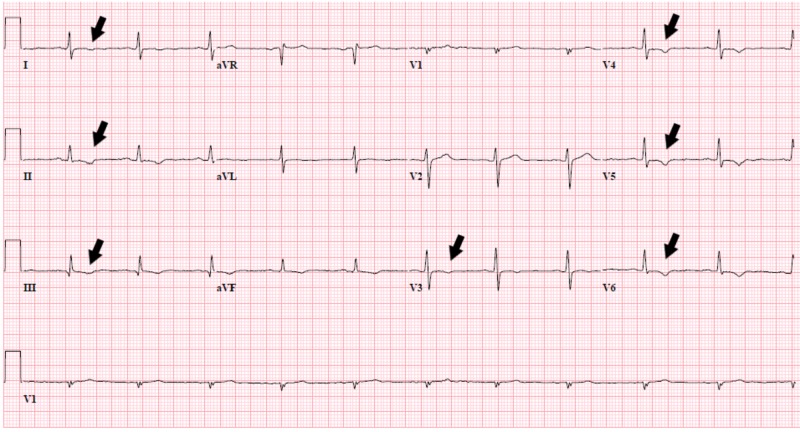
ECG with nonspecific T wave changes ECG: Electrocardiogram

Electrocardiogram (ECG) showed nonspecific T wave changes in inferior leads. Troponin levels were normal. Echocardiogram (ECHO) with an ejection fraction of 59% and normal diastolic left ventricular function. ECG changes were attributed to hypothyroidism (Figure 1).

**Figure 2:**
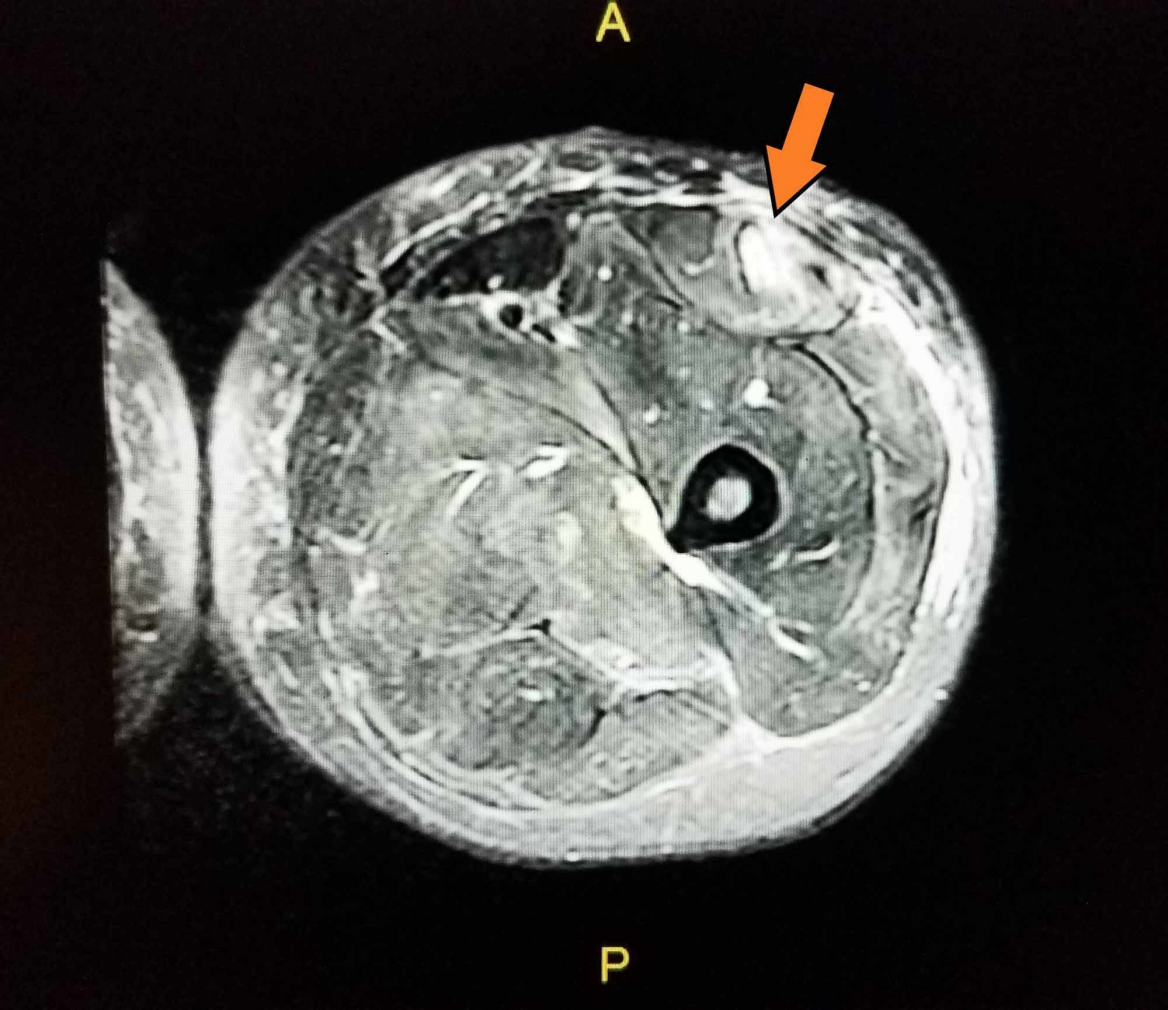
MRI of right thigh with an impaled piece of wood

Electroencephalogram (EEG) findings were abnormal indicating right hemisphere abnormality superimposed on diffuse brain dysfunction. This may have correlated with focal structural lesions or any combination of toxic, metabolic, infection or/and inflammatory etiologies. MRI of the brain without contrast did not show intracranial pathology. MRI of the right thigh with a piece of wood presented below (Figure 2).

Patient’s clinical condition significantly improved after treatment with intravenous levothyroxine, aggressive hydration, and pain management. He was given a total of four liters of normal saline with target urinary output of more than 35 ml/hr, his creatinine level returned to the baseline within two days and CK level decreased two-fold within four days. Regarding thyroid hormone replacement therapy, he was to be initiated on Levothyroxine 150 mcg intravenously daily but refused due to “feeling bad on such a high dose”. Finally, he was given Levothyroxine 100 mcg intramuscularly daily with decrease of TSH to 46.159 uIU/m and CK to 200s U/l upon discharge.

The patient was medically stabilized for the surgical debridement and undergone the procedure, which he tolerated well. After that, his home regimen of bi-weekly intramuscular levothyroxine was resumed, medication compliance was reinforced, and he was discharged in a stable condition.

## Discussion

Rhabdomyolysis is confirmed by elevated serum CK with minimum thresholds between 1000 and 10,000 U/L [3]. The normal range of CK levels is 45-260 U/L. Recurrent cases of rhabdomyolysis are quite rare as most patients experience only one episode of rhabdomyolysis in their lifetime. Most common causes of such episodes are medication, trauma or epileptic seizures. Further investigation is warranted if a patient with recurrent rhabdomyolysis has a family history of neuromuscular disorders or exercise intolerance [2].

The patient described in this case has had at least eight documented episodes of rhabdomyolysis associated with high TSH levels (see Table 1). However, he never followed up as outpatient to confirm his normal TSH, free T4 and CK levels on Levothyroxine therapy. We only have data on CK and TSH levels close to discharge when the patient was placed on regular levothyroxine injections and was definitely compliant with regimen (Table 2).

**Table 2:** Corresponding levels of TSH and CK during treatment TSH: Thyroid stimulating hormone; CK: Creatine kinase.

	Day 1	Day 3	Day 4	Day 5	Day 7	Day 9	Day 11
TSH	189.152	123.577	99.323	91.864	76.69	75.354	46.159
CK	10808	7171	5288	3952	2017	1191	401

Certainly, patient's seizure episodes and muscle trauma contributed to muscle breakdown. However, at the time of his presentation for surgical debridement he already had the trauma for three months and last seizure episode was two weeks prior to his presentation. We believe that muscle breakdown in this case also is associated with severe hypothyroidism due to poor medication compliance.

The severity of rhabdomyolysis may vary from asymptomatic rise in serum muscle enzymes to serious disease with several complications, including acute renal failure, metabolic derangements, disseminated intravascular coagulation, compartment syndrome, and peripheral neuropathy [1].

Acute kidney injury (AKI) is one of the most common complications with the reported frequency of AKI ranges from 15 to over 50% [4,5]. In critically ill patients, mortality rates reported as high as 59% [6]. Acute kidney injury is the most important cause of morbidity in patients with rhabdomyolysis.

In our case, the patient had multiple precipitating factors, such as muscle injury, polymyositis, and medication noncompliance which provoked the development of rhabdomyolysis. However, his case is interesting in that he had repeatedly presented on multiple admissions with markedly elevated creatine kinase levels and had never developed complications such as acute kidney injury, cardiac arrhythmias or severe electrolyte imbalances.

The concentration of CK in plasma correlates with the severity of rhabdomyolysis, and concentrations >5000 U/L is predictive of increased risk of AKI development [7]. Over the two years, the patient had visited our hospital 16 times (eight emergency room visits and eight hospital admissions) and on eight different occasions his serum CK was >5000 U/L (average 11599 U/L) with corresponding average creatinine level of 1.4 mg/dL (baseline 1.2 mg/dL).

In a retrospective analysis of patient’s hospital course over the last two years, we noted that he was always timely and aggressively hydrated with achieving adequate diuresis within the first 12-24 hours. The goals of fluid resuscitation for AKI prevention are dilution of toxins and achievement of adequate diuresis [8]. Usually, resuscitation with normal saline at a rate of 1-2 L/hr required to achieve urine output at least 0.6 ml/kg/hour. Once adequate diuresis is established, urine output should be maintained at 200 to 300 mL/hour. Fluid resuscitation should be continued until plasma CK levels decrease to <1000 unit/L [9]. Appropriate fluid resuscitation is the mainstay therapy for AKI prevention and should be initiated in a timely manner [10].

Thereby, an absence of AKI in this patient might be attributed to timely and aggressive hydration. At the same time, there are some studies showing weak correlation of serum CK levels with the risk [5, 11]. McMahon et al. conducted a large study in 2000 patients over a 10-year period and found no linear association between level of CK and risk of renal replacement therapy for AKI [11].

## Conclusions

This case describes a patient with recurrent uncomplicated rhabdomyolysis due to severe hypothyroidism. Rhabdomyolysis is rare and associated with several complications among which acute kidney injury is the most common. Appropriate fluid resuscitation is the mainstay therapy for AKI prevention and should be initiated in a timely manner. Recurrent rhabdomyolysis in a patient should prompt further investigation if there is a family history of a neuromuscular disorder. In a case of refractory hypothyroidism, a patient should be counseled on medication compliance and proper regimen.
